# RIPK3 and AXL Expression Study in Primary Cutaneous Melanoma Unmasks AXL as Predictor of Sentinel Node Metastasis: A Pilot Study

**DOI:** 10.3389/fonc.2021.728319

**Published:** 2021-10-21

**Authors:** Lorenzo Nicolè, Filippo Cappello, Rocco Cappellesso, Luisa Piccin, Laura Ventura, Vincenza Guzzardo, Paolo Del Fiore, Vanna Chiarion-Sileni, Angelo Paolo Dei Tos, Simone Mocellin, Ambrogio Fassina

**Affiliations:** ^1^ Department of Medicine (DIMED), University of Padova, Padova, Italy; ^2^ Unit of Surgical Pathology & Cytopathology, Ospedale dell’Angelo, Mestre, Italy; ^3^ Pathological Anatomy Unit, University Hospital of Padova, Padova, Italy; ^4^ Melanoma Oncology Unit, Istituto Oncologico Veneto (IOV-IRCCS), Padova, Italy; ^5^ Department of Statistical Sciences, University of Padova, Padova, Italy; ^6^ Soft-Tissue, Peritoneum and Melanoma Surgical Oncology Unit, IOV- IRCCS, Padua, Italy

**Keywords:** malignant melanoma, AXL, RIPK3, prognosis, metastasis

## Abstract

Malignant melanoma (MM) is the most lethal skin cancer. AXL is a tyrosine kinase receptor involved in several oncogenic processes and might play a role in blocking necroptosis (a regulated cell death mechanism) in MM through the downregulation of the necroptotic-related driver RIPK3. The aim of this study was to evaluate the clinical impact of the expression of AXL and RIPK3 in 108 primary cutaneous MMs. Association between AXL and RIPK3 immunoreactivity and clinical–pathological variables, sentinel lymph node status, and tumor-infiltrating lymphocytes (TILs) was assessed. Immunoreaction in tumor cells was detected in 30 cases (28%; range, 5%–80%) and in 17 cases (16%; range, 5%–50%) for AXL and RIPK3, respectively. Metastases in the sentinel lymph nodes were detected in 14 out of 61 patients, and these were associated with AXL-positive immunoreactivity in the primary tumor (p < 0.0001). No association between AXL and TILs was found. RIPK3 immunoreactivity was not associated with any variables. A final logistic regression analysis showed Breslow and AXL-positive immunoreactivity as the stronger predictor for positive sentinel node status [area under the receiver operating characteristic curve (AUC) of 0.96]. AXL could be a potential new biomarker for MM risk assessment, and it deserves to be further investigated in larger studies.

## Introduction

Malignant melanoma (MM) is the most fatal skin cancer, and its incidence is rising worldwide (1, 2). Screening activities, effective treatment of early-stage tumor, targeted therapy, and immune checkpoint inhibitors for metastatic disease (mMM) have significantly increased median and long-term survival; however, a subset of MMs still exhibits an aggressive behavior, with high rate of recurrence and/or short-lasting response to treatments (3–5).

Therefore, identification of new biomarkers is crucial for a better patient stratification and could possibly lead to the development of new therapeutic strategies.

AXL, along with TYRO3 and MERTK, is a member of the TAM family of receptor tyrosine kinases (6, 7). The main ligand of TAM receptors is the growth-arrest-specific protein 6 (GAS6). In cancer the Gas6/AXL signaling pathway is associated with tumor cell growth, metastasis, invasion, epithelial–mesenchymal transition (EMT), angiogenesis, drug resistance, immune regulation, and stem cell maintenance (8).

Overexpression of AXL has been described in different cancer types, and several therapeutic agents targeting AXL are currently under development (8–11).

In cutaneous MM, AXL expression has been correlated with higher cell mobility, invasive ability, and resistance to various targeted therapies (12–15). Furthermore, a recent study showed that elevated serum levels of the extracellular portion of AXL, which can enter the circulation after proteolytic cleavage by the proteases ADAM10 and ADAM17, correlates with disease progression and poor survival in cutaneous MM (7).

Recently, a study on cancer cell lines showed that AXL could be implicated in the inhibition of necroptosis (NCP), a form of programmed cell death, through downregulation of RIPK3 (16). NCP is driven by three main proteins, namely, RIPK1, RIPK3, and MLKL. NCP causes the rupture of the cell membrane, with consequent release of cell constituents into the extracellular environment (17, 18).

Through the release of cancer cells constituents, NCP may induce local inflammation, which impacts on the complex tumor ecosystem influencing both the biological behavior of cancer cells and the local immune response (18). RIPK3, a protein required in NCP, was found to be downregulated in MM, suggesting that inhibition of NCP could play a role on MM development and progression (16, 19–21).

Moreover, different studies showed that the tumor-infiltrating lymphocytes (TILs) and, more in general, the tumor-associated immune environment are involved in MM progression (22, 23). However, the impact of AXL and the other receptors of its family (TYRO3 and MERTK) in the regulation of the MM-associated immune environment has not been investigated to date, despite recent studies describing the expression of TAM receptors in MM (24).

Within this scenario, our objective was to assess AXL and RIPK3 expression in a retrospective series of primary cutaneous MM and to evaluate their association with clinicopathological variables, in order to explore their prognostic value. Second, we also investigated the impact of AXL and RIPK3 on TILs.

## Materials and Methods

### Patients

The study was carried out on formalin-fixed and paraffin-embedded (FFPE) surgical specimens of 108 cutaneous malignant melanomas resected in the period 1998–2011. The specimens were retrieved from the archives of the Surgical Pathology and Cytopathology Unit of the University of Padova. The inclusion criteria were as follows:

1. availability of adequate surgical specimen;
2. tumors located in trunk or limbs [non-chronically sun-damaged (CSD) areas];
3. availability of follow-up data; and
4. no previous treatment before surgery.

All the experimental procedures were performed according to the 1964 Helsinki declaration and its later amendments. This study follows the REporting recommendations for tumor MARKer prognostic studies (REMARK) guidelines (25).

### Immunohistochemistry

Immunohistochemical studies were conducted on 4-μm-thick sections obtained from each FFPE tissue sample. Staining was done using the BOND Polymer Refine Detection kit (Leica Biosystems, Newcastle Upon Tyne, UK) in the BOND-MAX system (Leica Biosystems) as described elsewhere (26).

**Figure 1:**
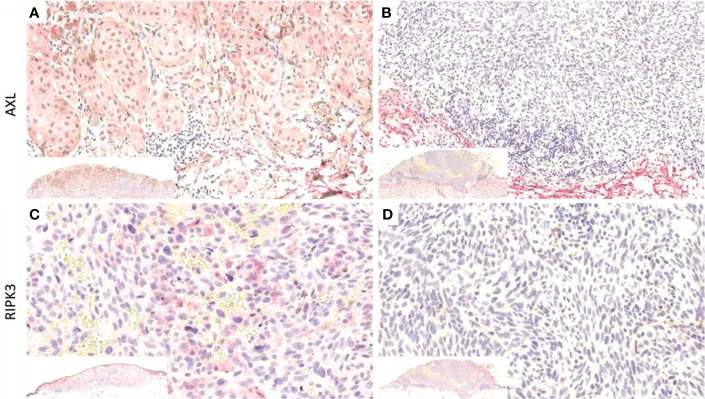
Representative cases showing immunoreaction for AXL **(A)** and RIPK3 **(C)** immunostain, and cases with no immunoreaction for AXL **(B)** and RIPK3 **(D)** immunostain. In the inner box on the low left of each picture, the correspondent case is shown. **(B, C)** Some case. Original magnification, 200×.

Primary antibodies and clones are listed in **Table 1**. Following the manufacturer’s suggestions, appropriate positive (human tonsil tissue for RIPK3 and human testis tissue for AXL) and negative (serum without the primary antibody) controls were run concurrently during analysis. RIPK3 and AXL reactions were scored as the percentage of positive tumor cells (TCs). Cytoplasmic or membrane immunostains were considered. All reactions were evaluated independently by two pathologists (LN and FC); the rare ambiguous or discordant scores were assessed collectively with a third pathologist (RC), in order to reach an agreement (**Figure 1**).

**Table 1:** Antibodies used for immunohistochemistry.

Antigen	Clone	Source	Vendor	Dilution
AXL	nbp1-83073	Rabbit polyclonal	Novus Biological	1:100
RIPK3	780115	Mouse monoclonal	R&D System	1:300
CD3	LN10	Mouse monoclonal	Leica Biosystems	1:100

### Tumor-Infiltrating Lymphocytes

**Figure 2:**
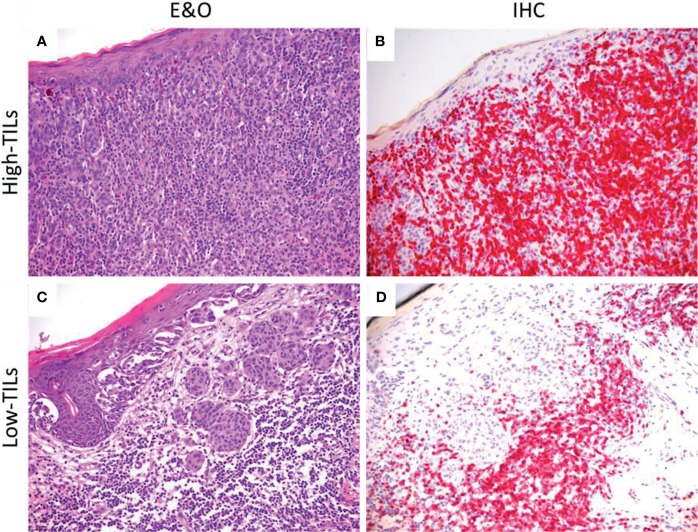
Hematoxylin and eosin staining (left) and CD3 immunohistochemistry (right) demonstrating different degrees of lymphocytic infiltration. **(A, B)** Melanoma with marked lymphocytic infiltration, here classified as “high TILs”. **(C, D)** Melanoma with lymphocytes surrounding but not infiltrating tumor cells, here classified as “low TILs”. **(A–C)** Hematoxylin and eosin staining (E&O); original magnification, 100×. **(B–D)** Immunostaining (IHC) for CD3; original magnification, 100×.

We determined lymphocytic infiltrate through CD3 immunostain. We considered areas of lymphocytic infiltration only those in which TILs could be detected both in hematoxylin and eosin and in CD3-stained sections. Only lymphocytes that were in direct contact with melanoma cells and disrupted tumor nests were retained for scoring. Cases were dichotomized in “low TILs” if the infiltration was absent or non-brisk and “high TILs” if the infiltration was brisk, according to the definitions given by Lee et al. (27). All samples were jointly evaluated by two pathologists (LN and FC) who were unaware of any clinical information (**Figure 2**).

### Statistical Analysis

Categorical variables were expressed as frequencies (and percentages) and numerical variables as means (and standard deviations). The correlation between continuous variables was computed using Pearson’s correlation. Contingency tables were analyzed using Fisher’s exact test. Differences in quantitative variables between subject groups were assessed with Student’s t-test or Mann–Whitney non-parametric test, according to the Shapiro–Wilk test of normality. Survival curves were estimated with the Kaplan–Meier method and were compared with the log-rank test. A Cox proportional hazards (HR) regression model was used both to estimate the HRs and for a multivariate survival analysis. Finally, multiple logistic regression was used to model the two categories of sentinel lymph node (SLN) status according to the available covariate. The receiver operating characteristic (ROC) curve, with the corresponding area under the ROC curve (AUC), and the Hosmer–Lemeshow goodness of fit test were performed to validate the fitted logistic regression. The ROC curve was also used to assess Axl and Breslow score as sentinel nodes status predictors, and the Youden criterion was applied to compute an optimal cutoff for AXL. The free-software R (http://www.r-project.org/) was used for statistical analyses. The level of significance was set at p < 0.05.

## Results

### Clinicopathological and Immunohistochemical Characteristics

The demographic and the main clinicopathological parameters of patients recruited for this study are summarized in **Table 2**.

**Table 2:** Patients’ clinicopathological characteristics.

Characteristics	Number of patients (percentages)
Age at diagnosis in years, mean ± SD (min–max)	61.62 ± 14.11 (33–95)
Sex, n (%)	
▪ Female	41 (37.96)
▪ Male	67 (62.04)
Tumor thickness in mm, n (%)	
▪ ≤1	77 (71.30)
▪ 1.01–2	19 (17.59)
▪ > 2.01– 4	12 (11.11)
Clark’s level, n (%)	
▪ II	23 (21.30)
▪ III	46 (42.59)
▪ IV	37 (34.26)
▪ V	2 (1.85)
SLN status, n (%)	
▪ Negative	47 (43.52)
▪ Not evaluated	47 (43.52)
▪ Positive	14 (12.96)
Stage	
▪ IA	77 (71.29)
▪ IB	9 (8.33)
▪ IIA	7 (6.48)
▪ IIB	3 (2.77)
▪ IIIA	6 (5.55)
▪ IIIB	3 (2.77)
▪ IIIC	3 (2.77)
AXL	
▪ Negative	91 (82.26)
▪ Positive	17 (15.74)
TILs	
▪ High	18 (16.67)
▪ Low	90 (83.33)

A total of 108 patients were included in our study (67 men and 41 women; mean age, 61.6 ± 14.11 years; range, 33–95 years). Most of the tumors (n = 86, 79.63%) were in stage I at diagnosis, 10 (9.26%) were in stage II, and 12 (11.11%) in stage III, according to the eighth edition of the American Joint Committee on Cancer (AJCC) staging system (28). Sentinel lymph nodes (SLNs) were positive in 14 cases (12.96%) and negative in 47 cases (43.52%); in the remaining 47 cases (43.52%), SLN status was not evaluated. Overall, 17 cases (15.74%) showed positive immunoreaction for AXL and 3 cases (2.78%) for RIPK3. In 18 tumors (16.67%), the lymphocytic infiltration was categorized as “high TILs”, while in the other 90 cases (83.33%), it was classified as “low TILs”. The median follow-up was 112.5 months (range, 1.0–186.2 months).

### Association Between AXL and RIPK3 Expression With Clinicopathological Characteristics

Eleven of the 17 cases (64.70%) with positive immunoreaction for AXL had also positive for SLN status. Only one of the AXL-positive cases (2.13%) had negative SLN. In the remaining five AXL-positive cases (10.64%), SLN status was not evaluated. Considering only the 61 cases in which SLN was examined, AXL-positive immunoreactivity was significantly associated with the presence of nodal metastasis (p < 0.001). Considering the stage, 96 cases (98%) were stage I or II according to the TNM AJCC staging system, 8th edition, while the remaining 12 cases (2%) were stage III or IV. Only eight cases (8%) belonging to the combined stage groups I and II were AXL positive. In the group combining stage III and IV, nine cases (75%) resulted positive for AXL immunoreaction. AXL-positive immunoreaction was strongly different between early stage (combined stage I and II) and advanced stage (combined stage III and IV), p < 0.001.

RIPK3 immunoreactivity was found in only 17 cases (16%; range, 5%–50%). No significant association between RIPK3 expression and clinicopathological variables was found.

### AXL and Breslow Score as Sentinel Nodes Status Predictors

**Figure 3:**
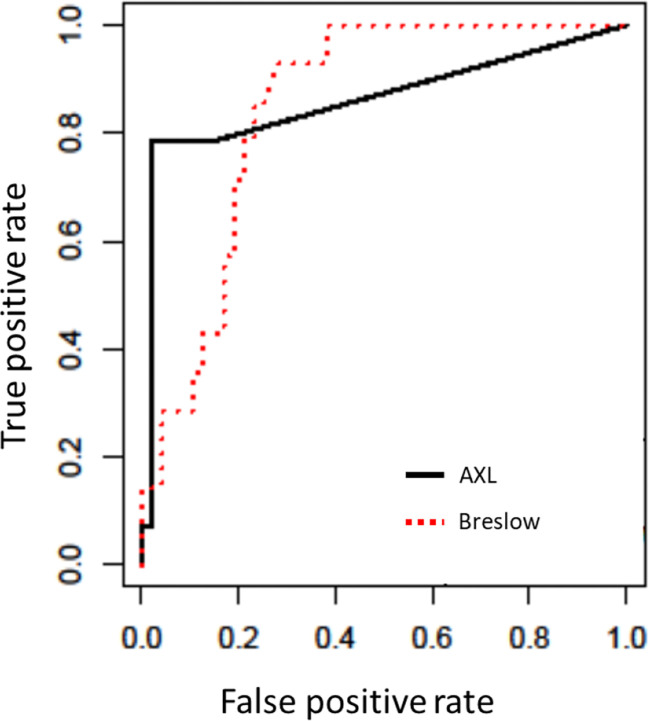
Receiver-operating characteristic (ROC) curves obtained for AXL and Breslow index. Area under the curve was 0.86 for AXL and 0.85 for Breslow index.

The possible association of AXL and RIPK3 immunoreaction with nodal metastasis was investigated in the 61 cases with known SLN status. For AXL, the Youden criterion identified 27.5 as the best threshold to discriminate between patients with positive SLN status *versus* negative. The ROC curve for AXL (**Figure 3**) presented an AUC of 0.86 (95% CI, 0.73–0.99). Overall, 57 out of 61 cases were correctly classified with a sensitivity of 0.79 (95% CI, 0.69–0.89) and a specificity of 0.98 (95% CI, 0.94–1.00). The ROC curve for Breslow (**Figure 3**) presented an AUC of 0.85 (95% CI, 0.75–0.94). Overall, with the threshold 0.8, 47 of 61 cases were correctly classified with a sensitivity of 0.93 (95% CI, 0.87–0.99) and a specificity of 0.72 (95% CI, 0.61–0.83).

### Survival Analysis

Stage I and II MMs were associated with a longer overall survival if compared with stage III tumors (p < 0.001; **Table 3**). Tumor thickness (dividing the patients into four categories based on the T parameter of the TNM staging system: T1, T2, T3, and T4) was significantly associated with OS (p < 0.001).

**Table 3:** HR (and 95% CI) for overall survival and p-value of the log-rank test.

		N	HR (95% CI)	p-Value
Gender	Female	41	Ref	
	Male	67	3.78 (1.11;12.93)	**0.035**
Breslow	≤1	77	Ref	
	1.01–2	19	4.98 (1.51;16.42)	**0.008**
	>2.01–4	12	23.71 (7.71;72.95)	**<0.0001**
Clark’s level	II	23	Ref	
	III	46	1.20 (0.23;6.22)	0.82
	IV–V	37	4.14 (0.93;18.46)	0.06
SLN status	Negative	47	Ref	
	Positive	14	12.01 (4.12;34.96)	**<0.0001**
Stage	I–II	96	Ref	
	III–IV	12	7.98 (3.19;19.94)	**<0.0001**
TILs	High	18	Ref	
	Low	90	0.22 (0.03;1.66)	0.14

N, Number of patients; HR, Hazard Ratio; CI, confidence interval.

Bold values correspond to significant value.

Cox multivariate analysis using a backward stepwise selection method revealed Breslow score to be the sole predictors of survival. The parameters significant at multivariate survival analysis were for Breslow score HR = 2.36 (95% CI = 1.69–3.31), p < 0.0001 (**Table 4**). The test for the proportional hazards assumption validated the Cox regression model fit (p = 0.37).

**Table 4:** OR and p-value for overall survival of the Cox multivariate analysis.

	p-Value	OR	OR lower	OR high
Breslow	**0.004**	2.01	1.34	3.66
Stage	**<0.001**	164.0	22.19	2980.9
Clark’s Level	0.25	1.85	0.67	5.47
TILs	0.14	0.2	0.01	1.17

OR, Odds Ratio.

Bold values correspond to significant value.

### Multiple Regression Logistic Analysis

**Figure 4:**
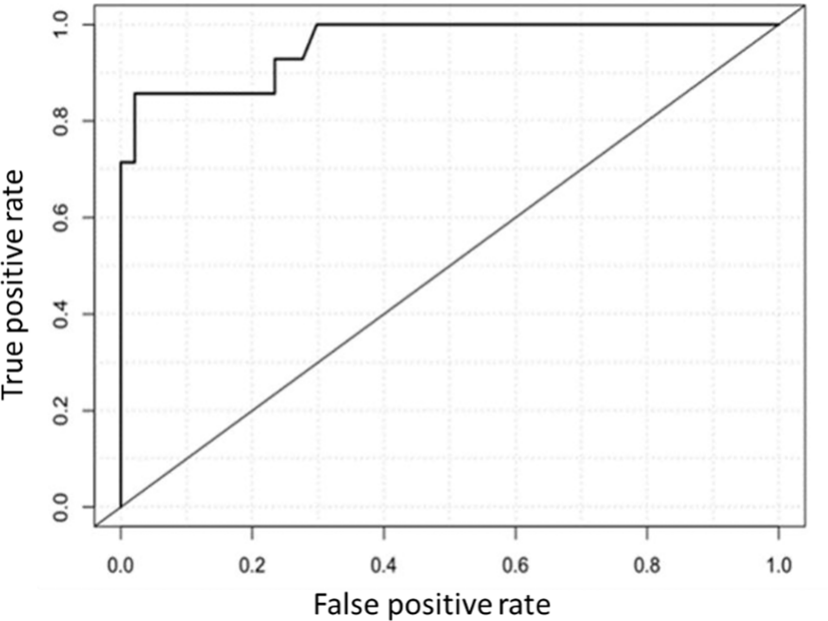
Receiver-operating characteristic (ROC) curve for prediction of sentinel lymph node status through a logistic model based on AXL expression and Breslow score as significant covariates. The area under the curve is 0.96.

A logistic regression analysis was performed to model SLN status using a backward stepwise selection method. The resulting fitted model showed as significant covariates AXL [log(OR) = 5.13 ± 1.28, p < 0.0001] and Breslow score [log(OR) = 0.78 ± 0.36, p = 0.031]. The receiver operating characteristic curve is shown in **Figure 4**, and the AUC is 0.96 (95% CI, 0.85–1.00). The Hosmer–Lemeshow goodness-of-fit test validated the model (p = 0.68). Overall, 58 of 61 (95%) cases were correctly classified with a sensitivity of 0.86 (95% CI, 0.57–0.98), a specificity of 0.98 (95% CI, 0.89–1.00), a positive predictive value of 0.92 (95% CI, 0.64–1.00), a negative predictive value of 0.96 (95% CI, 0.86–0.99), a positive likelihood ratio of 40.29 (95% CI, 5.73–283.37) and a negative likelihood ratio of 0.15 (95% CI, 0.04–0.53).

## Discussion

Metastatic MM is one of the most lethal cancers worldwide. During the last decades, the spreading of prevention strategies and the progresses in treatment have increased survival. However, prognostic stratification at diagnosis remains challenging. To further improve the clinical management of MM, it is crucial to identify patients with a high risk of progression, especially among those with early-stage tumors.

Positive SLN status, including the spatial distribution of the tumor burden within the lymph node, remains the best prognostic factors available so far (29).

According to the current international guidelines (30, 31), SLN examination is performed in patients with MM staged at least as T1b according to the AJCC staging system, while there is no a consensus on which patients below the T1b are offered for SLN examination. SLN positivity rate increases with the primary tumor thickness (measured as Breslow index) that, to date, remain the more common marker to manage the risk assessment for lymph node metastasis (32). However, a recent meta-analysis reported an overall rate of only 5% of SLN metastases in patients with thin MM (Breslow thickness <1 mm), highlighting the need to better define the selection criteria for lymph node examination in thin MM (33). Tejera-Vaquerizo et al. investigated 4,249 thin MM (thickness of 1 mm or less) showing that only higher mitotic rate, >2 mitoses/mm^2^, after multivariate analysis resulted as the best significant SLN-positive predictor with an odds ratio (OR) of 2.9 (95% CI, 1.22–7) (34). In line with Tejera-Vaquerizo, also the study of Egger et al. showed higher mitoses rate significant in predict SLN-positive status OR of 2.01 (95% CI, 1.54–2.34) (35). In a further larger study with more than 12,000 patients, Egger et al. confirmed the aforementioned results, introducing also younger age and lymph vascular invasion as significant SLN-positive markers in patients with T2 MM, underlying as Breslow index alone could potentially lead to overtreatment of patients (36). Moreover, another larger retrospective study showed that the age with a cutoff at 55 years is a significant marker to predict SLN status, especially in thin melanoma (37). Despite results showing that parameters such as mitosis count, age, and lympho-vascular invasion can improve the accuracy of patients stratification when associated to the Breslow index, nomogram comprising these markers is not still largely used in clinical practice or encouraged by expert guidelines.

Several studies also proposed gene expression profiling to stratify patients according to the risk for positive SLN. The more diffuse multigenes panel described so far is the so-called DecisionDx-Melanoma signature, a proprietary gene expression profile test (31-GEP), which involves a predictive modeling algorithm that determines whether the genetic profile of the tumor is more strongly associated with low or high risk to have positive SLN. Despite encouraging results, after prospective validation and meta-analytic studies, the use of molecular profiling of MM to stratify the metastasis risk is much more efficient in stratifying stage III patients with respect to thin MM (38).

Some studies reported TILs as an independent factor to predict SLN status; however, classical TIL assessment is challenging due to its subjective nature and low reproducibility among pathologists (22).

Within this scenario, new biomarkers to stratify patients according to their risk of regional nodal metastases are needed, in particular for patients with thin MM. In addition to morphological markers, immunophenotypic markers could be useful to implement models for the risk assessment.

In this study, the immunohistochemical expression of RIPK3 and AXL was assessed in a cohort of 108 patients with primary cutaneous MM in order to investigate the correlation of these two markers with clinicopathological features, with main regards to SLN status and TILs.

To date, a cutoff for the immunohistochemical evaluation of AXL and RIPK3 has not yet been proposed in MM. Due to the low percentage of cases with tumor cells with positive immunoreaction for RIPK3, the ROC and AUC for this marker have not been calculated, while for AXL, an exploratory cutoff of 27.5% of positive tumor cells has been calculated with the Youden criteria.

Considering the limitations due to the low number of patients investigated (if compared with studies mentioned above) and the impossibility to stratify our patients according to T stage, our results support AXL immunohistochemical evaluation as possible marker for the prediction of SLN status, especially if combined with Breslow index. Indeed, considering AXL expression and Breslow thickness within a logistic regression model, the predictive ability of SLN status increases, suggesting the inclusion of AXL evaluation in the MM diagnostic process. However, larger validation studies are needed to confirm this finding.

Observations that AXL expression leads to an increased risk for metastatic diseases is supported by previous works showing that AXL-mediated pathways in MM may drive and sustain cell migration, invasion, and drug resistance (3, 4, 39). Recently, AXL activity in MM was discovered to be driven by the transcriptional complex SOX2-GLI1 through the sialyltransferase ST3GAL1, which was found to be overexpressed in MM compared to nevi, and in metastatic MM compared to primary MM (14). Moreover, AXL and the other TAM receptors (TYRO3 and MERTK) have been reported as key mediator of chemoresistance in neuroblastoma through induction of EMT and in lung cancer through the regulation of mitogen-activated protein kinase (MAPK) and FAS signaling pathways (40). Finally, AXL was shown to be involved also in the immune regulation, thus having a potential role in mediating resistance to immune-checkpoint inhibitors or in creating an immunosuppressive tumor microenvironment permissive to tumorigenesis (41). All these findings suggest that AXL could be a possible therapeutic target. Many AXL-targeted agents have already been developed so far (8), and *in vitro* testing of antibodies directed against AXL in combination with immune checkpoint blockade has been carried out with encouraging results in MM (42).

Since previous studies reported a role of AXL in regulating necroptosis through RIPK3 (16) and described loss of RIPK3 during MM progression (20, 21), immunoreaction of RIPK3 was also evaluated in this study. In our cohort, only three MM (2.78%) retained RIPK3, and no association with AXL stain was observed. Taken together, these observations seem to suggest that downregulation of necroptosis is a common phenomenon in MM. The inhibition of necroptosis through downregulation of its main driver (RIPK1, RIPK3, and MLKL) has been observed in different cancer types, showing in some cases also a negative impact on patient survival (43). However, further studies using more sensitive methods are necessary to better evaluate necroptosis in MM. Moreover, since the impact of necroptosis in cancer biology resides in its link with the immune environment, it could be useful to set up studies that consider more deeply necroptosis and its association with the different immune elements associated with the tumor (44). In this study, the evaluation of the immune environment was limited to lymphocytes, and neither AXL or RIPK3 showed association with TILs. Moreover, in our series, a higher TIL level was not associated with increased OS, as reported in other studies (22).

Moreover, in our cohort, AXL-positive immunoreaction was found in only five patients with stage I. Contrarily, most of the cases with advanced stage (at least IIIA) showed high frequency of AXL positivity.

Although the promising results and the several strengths of this study, such as the high rate of patients with SLN evaluation, some limitations must be pointed out. First, the retrospective design of the study does not consider aspects that could affect the OS and SLN status, such as pharmacological therapy or comorbidity. Second, our study is limited to a cohort containing only patients with non-CSD MM, thus excluding all the other types of MM as CSD-associated, acral, mucosal, or uveal MM. Considering the different genetic background that featured each type of MM, studies on larger cohorts of patients covering all the types are needed to test the validity of our observations. Moreover, the mutational status of the patients recruited for this study was largely unknown, thus preventing any evaluation of its possible association with AXL or RIPK3 expression. Third, the investigation was carried out only with immunohistochemistry, without the use of more sensitive methods, such as reverse transcription PCR (RT-PCR), for direct quantification of AXL and RIPK3 mRNA.

This study identified for the first time a potential role of AXL, especially if combined with Breslow index, to improve the accuracy of SLN status prediction in primary cutaneous MM. Considering the explorative design of this study, further studies are needed to validate our results. Moreover, further study analyzing gene and protein expression with more sensitive methods will be carried out to determine whether the inhibition of necroptosis through loss of RIPK3 represents a frequent event in MM. A prospective study will also be designed in order to better evaluate the role of AXL in MM management and its real impact on disease progression and patient survival.

## Data Availability Statement

The original contributions presented in the study are included in the article/**Supplementary Material**. Further inquiries can be directed to the corresponding author.

## Ethics Statement

Ethical review and approval was not required for the study on human participants in accordance with the local legislation and institutional requirements. Written informed consent for participation was not required for this study in accordance with the national legislation and the institutional requirements.

## Author Contributions

Conceptualization: LN, AF, and FC. Methodology: LN, FC, and RC. Analysis: LV. Investigation: LN, FC, and VG. Data curation: LN, FC, and PF. Writing—original draft and preparation: LN, RC, LV, and FC. Writing—review and editing: LN, RC, FC, LP, VC-S, and AD. Supervision: AF and SM. Funding acquisition: AF and SM. All authors contributed to the article and approved the submitted version.

## Conflict of Interest

The authors declare that the research was conducted in the absence of any commercial or financial relationships that could be construed as a potential conflict of interest.

## Publisher’s Note

All claims expressed in this article are solely those of the authors and do not necessarily represent those of their affiliated organizations, or those of the publisher, the editors and the reviewers. Any product that may be evaluated in this article, or claim that may be made by its manufacturer, is not guaranteed or endorsed by the publisher.

## Supplementary Material

The Supplementary Material for this article can be found online at: https://www.frontiersin.org/articles/10.3389/fonc.2021.728319/full#supplementary-material
